# Inhibition of neurogenic contractions in renal arteries and of cholinergic contractions in coronary arteries by the presumed inhibitor of ADP-ribosylation factor 6, NAV2729

**DOI:** 10.1007/s00210-022-02218-2

**Published:** 2022-02-10

**Authors:** Ru Huang, Bingsheng Li, Alexander Tamalunas, Raphaela Waidelich, Christian G. Stief, Martin Hennenberg

**Affiliations:** 1 Department of Urology, University Hospital, LMU Munich, Munich, Germany; 2 Urologische Klinik Und Poliklinik, Marchioninistr. 15, 81377 Munich, Germany

**Keywords:** NAV2729, ARF6, Vascular smooth muscle, Vasocontraction

## Abstract

NAV2729 is a presumed inhibitor of the monomeric GTPase ADP ribosylation factor 6 (ARF6) and inhibits smooth muscle contraction outside the cardiovascular system. Its effects on vascular smooth muscle contraction or a possible role of ARF6 in vasocontraction have not yet been examined. Here, we report effects of NAV2729 on neurogenic and agonist-induced contractions in renal interlobar and coronary arteries. Contractions of pig interlobar and coronary arteries were induced in an organ bath by agonists or by electric field stimulation (EFS). Owing to divergent characteristics of both vessel types, EFS-induced contractions were only examined in interlobar arteries, and contractions by agonists acting on muscarinic receptors only in coronary arteries. NAV2729 inhibited frequency-dependent EFS-induced contractions of interlobar arteries. The degree of inhibition was similar using 5 µM and 10 µM NAV2729. Inhibition of EFS-induced contractions was resistant to a nitric oxide synthase inhibitor and to diclofenac. The neurogenic and adrenergic character of EFS-induced contractions was confirmed by inhibition by tetrodotoxin and prazosin. In coronary arteries, NAV2729 (5 µM) inhibited concentration-dependent contractions induced by carbachol and methacholine. Contractions induced by α_1_-adrenergic agonists, endothelin-1, the thromboxane receptor agonist U46619, or serotonin remained unchanged by NAV2729 in both vessel types. NAV2729 inhibits neurogenic contractions in interlobar arteries and contractions induced by cholinergic agonists in coronary arteries. In both vessel types, NAV2729 does not inhibit contractions induced by receptor agonists other than those acting on muscarinic receptors. Addressing effects in other vessels and in other smooth muscle–rich organs merits further attention.

**Supplementary Information:**

The online version contains supplementary material available at 10.1007/s00210-022-02218-2.

## Introduction

ADP ribosylation factor 6 (ARF6) belongs to the superfamily of monomeric GTPases. Major known functions of ARF6 encompass promotion of actin polymerization, roles in vesicular trafficking, regulation of internalized cargo, cell adhesion, migration, and completion of mitotic cytokinesis (D'Souza-Schorey and Chavrier 2006; Donaldson 2002; Humphreys et al. 2013; Schafer et al. 2000; Schweitzer and D'Souza-Schorey 2005; Van Acker et al. 2019). Based on evidence obtained by knockout of ARF6 and using NAV2729, a small molecule inhibitor with presumed specificity for ARF6, ARF6-mediated promotion of prostate smooth muscle contraction has been suggested (Wang et al. 2021; Yu et al. 2019). A panel of intracellular mediators of smooth muscle contraction is shared by different organs, including the monomeric GTPase RhoA, which promotes contractions in any smooth muscle–rich organ. Meanwhile, similar roles of other monomeric GTPases in smooth muscle contraction are increasingly emerging (Li et al. 2020). Consequently, a role of ARF6 in smooth muscle contraction may not be limited to the prostate and addressing the effects of NAV2729 in other smooth muscle types merits investigation.

Acknowledging the role of vascular smooth muscle contraction for cardiovascular homeostasis and diseases, together with their high prevalence, burden, and mortality, it might be adequate to assess effects of NAV2729 or to understand a driving role of ARF6 in vascular smooth muscle contraction. Diseases and conditions imparted by aberrant vascular smooth muscle contraction include arterial hypertension, coronary artery disease, diabetic nephropathy, and others. Globally, elevated systolic blood pressure has been estimated to account for 7.7 to 10.4 million annual deaths (Zhou et al. 2021), which is outnumbered by the number of deaths due to any cardiovascular disease, including ischemic heart disease, estimated to 18.6 million cases for the year 2019 (Roth et al. 2020). Drugs to inhibit vascular smooth muscle contraction belong to the gold standard options in medical treatment of hypertension and cardiovascular diseases (Brouwers et al. 2021). Diabetic nephropathy represents a major complication of diabetes, affecting 17–35% of patients with diabetes and accounting for 50% of patients entering dialysis or programs for kidney transplantation in the USA (DeFronzo et al. 2021; Lin et al. 2021). Impaired renal function in diabetic nephropathy is related to increased glomerular capillary pressure and increased smooth muscle tone in efferent glomerular arterioles, which, in turn, is closely coupled to systemic blood pressure and hypertension (DeFronzo et al. 2021; Khavandi et al. 2009). Accordingly, strategies for treatment of diabetic nephropathy include drugs to reduce intrarenal vascular resistance and antihypertensive drugs, in addition to glucose-lowering sodium-glucose cotransporter 2 (SGLT2) inhibitors (Cosentino et al. 2020).

Vascular smooth muscle contraction is induced after activation of G protein–coupled receptors by contractile agonists, including sympathetic neurotransmitters (noradrenaline, serotonin), humoral factors (endothelin-1), and paracrine mediators (thromboxane A_2_) in most vessel types, but also cholinergic activation of muscarinic receptors in coronary arteries (Liu and Khalil 2018; Touyz et al. 2018). Receptor activation results in activation of at least three intracellular signaling cascades, which are shared by smooth muscle in any smooth muscle–rich organ and include calcium-dependent activation of myosin light chain kinase, paralleled by calcium sensitization via protein kinase C and the RhoA/Rho kinase pathway (Hennenberg et al. 2014, 2008; Somlyo and Somlyo 2003). Functions of monomeric GTPases other than RhoA in smooth muscle contraction become increasingly obvious (Li et al. 2020).

Following our recent findings that NAV2729 inhibits and ARF6 promotes smooth muscle contraction in the prostate (Wang et al. 2021; Yu et al. 2019), we were interested whether similar effects of NAV2729 may be observed in vascular smooth muscle contraction. In this pilot study, we examined effects of NAV2729 on contraction of isolated pig renal and coronary arteries.

## Materials and methods

### Pig arteries

Pigs were sacrificed for meat production in a slaughterhouse at night. Kidneys and hearts were fetched up by a butcher still at night-time, transported to the butcher’s shop (Metzgerei Brehm, Planegg, Germany) (transport and subsequent storage at 4 °C), and from there, transferred to the laboratory in the morning. Preparation of interlobar arteries from kidneys and of middle sections of left anterior descending arteries was started immediately after arrival of organs in the laboratory. Adipose and connective tissues were removed from dissected arteries, and vessels were cut into rings, which were stored in Custodiol^®^ solution (Köhler, Bensheim, Germany) at 4 °C until being used. Experiments were started within 3 h after vessel preparation. Diameters of renal interlobar arteries ranged between 3 and 4 mm and around 5 mm for coronary arteries.

### Organ bath studies

From prepared vessels, rings with a length of 2–3 mm were cut, which were mounted in chambers of an organ bath system (model 720 M, Danish Myo Technology, Aarhus, Denmark). The organ bath device includes four channels, which were filled with rings from the same artery, filled with 10 ml aerated (95% O_2_ and 5% CO_2_) Krebs–Henseleit solution (37 °C, pH 7.4), and examined simultaneously. Following mounting, rings from interlobar arteries were stretched to 9.8 mN, while rings from coronary arteries were stretched to 19.6 mN for pretension. Typically, pretensions decline spontaneously after mounting, so that tensions were adjusted three times, resulting in stable, designated pretensions of 9.8 mN or 19.6 mN, respectively, after 45 min. Subsequently, a 2 M KCl solution was added to the Krebs–Henseleit solution in organ bath chambers, to obtain a potassium concentration of 80 mM in a hyperosmolar manner, in order to assess high-molar KCl-induced contractions as a measure for tissue size and smooth muscle content and condition, and for later reference of agonist-induced and neurogenic contractions. Once a plateau or maximum contraction was obviously obtained, high-molar KCl was rinsed by washing all chambers three times with Krebs–Henseleit solution within 30 min. Subsequently, NAV2729, tetrodotoxin (TTX), prazosin, or equivalent amounts of solvent (for controls, dimethylsulfoxide (DMSO) for NAV2729 and prazosin, water for TTX) were added. Thirty minutes after the addition of drugs or solvent, frequency response curves by electric field stimulation (EFS) or cumulative concentration response curves for agonists were constructed. In interlobar arteries, application of EFS results in contractions by release of endogenous neurotransmitters, due to stimulation of action potentials in residual neurons in examined tissues. NAV2729 was applied in final concentrations of 5 µM, 10 µM, or 20 µM and was added as a stock solution with a concentration of 10 mM.

From four channels being filled with rings from the same artery, two were examined with drug (NAV2729, TTX, or prazosin), and two others with solvent for one independent experiment. Only one frequency response or concentration response curve was recorded with each vessel ring. Allocations of drug and control channels were changed between different experiments. Independent experiments were repeated in indicated numbers (*n*), using vessels from *n* different animals, resulting in numbers of independent experiments as indicated for each series. Accordingly, drug and control groups were examined in the same experiments and obtained using the same vessels in each series. Single experiments were based on double determinations wherever this was possible. From a total of 64 organ bath experiments performed with renal arteries, double determination (i.e., including two technical replicates) was possible in 51 experiments for control groups and in 58 experiments for drugs (NAV2729 or prazosin) (see supplementary information files). From a total of 42 organ bath experiments performed with coronary arteries, this was possible in 40 experiments for control groups and in 33 experiments for NAV2729 (see supplementary information files). Data from remaining experiments, where double determinations were not possible, are based on single technical replicates, which were otherwise technically successful.

For reporting of EFS- and agonist-induced contractions, tensions were calculated as percentage of 80 mM KCl-induced contractions, to correct for variabilities between tissues. Reference to KCl allows to visualize changes in receptor responsiveness, while correlations between force generation and tissue length, diameter, or weight are poor or lacking in organ bath experiments using vessel rings (Erdogan et al. 2020). As contractions by high-molar KCl were induced by the addition of a 2 M KCl solution to normal Krebs–Henseleit solution in organ bath chambers, a separate set of control experiments was performed to estimate possible contributions from elevated chloride concentrations due to the hyperosmolar addition of KCl. In these experiments, vessels were first exposed to additional 80 mM NaCl (i.e., by the addition of a 2 M solution to normal Krebs–Henseleit solution in organ bath chambers), followed by washout and assessment of KCl-induced contractions as described above. To assess effects of DMSO and NAV2729 on KCl-induced contractions in another series of experiments, contractions by KCl were induced before and after application of DMSO or NAV2729 (5 µM). Thus, after a first KCl-induced contraction and washout, either DMSO or NAV2729 was applied, and a second KCl-induced contraction was induced 30 min later. Finally, the second KCl-induced contraction was expressed as percentage of the first KCl-induced contraction.

In series, where frequency or concentration response curves provided obvious reasons to assume drug effects on *E*_max_ values, pEC_50_ values, or frequencies (*f*) inducing 50% of maximum EFS-induced contraction (Ef_50_ values), these values were calculated by curve fitting using GraphPad Prism 6 (Statcon, Witzenhausen, Germany). As a presentation of all single values in scatter plots was aimed, automatic curve fitting was performed separately for all single experiments, resulting in separate values from each independent experiment. Fitting of sigmoidal concentration response curves by nonlinear regression was performed using the same settings as previously reported (Li et al. 2021). As recommended in the “GraphPad Curve Fitting Guide” (GraphPad Software, Inc., San Diego, CA, USA), resulting values were checked for plausibility. In fact, obvious outliers occurred but were not removed and handled as follows. Ef_50_ and *E*_max_ values for EFS-induced contractions in one out of all experiments using EFS were marked as “ambiguous” by the program, as contractions were not (control group) or barely (NAV2729 group) frequency-dependent in this experiment. Consequently, Ef_50_ values of this experiment represent outliers but were still reported in scatter plots and included in calculations of means.

### Materials, drugs, and nomenclature

NAV2729 (3-(4-chlorophenyl)-5-(4-nitrophenyl)-2-(phenylmethyl)pyrazolo[1,5-a]pyrimidin-7(4H)-one) is a small molecule inhibitor with assumed selectivity for ARF6 (Yamauchi et al. 2017; Yoo et al. 2016). Prazosin (1-(4-amino-6,7-dimethoxy-2-quinazolinyl)-4-(2-furanylcarbonyl)piperazine) is a subtype-unselective α_1_-adrenoceptor antagonist (Alexander et al. 2021a). NAV2729 and prazosin were dissolved in DMSO (10 mM for NAV272, 0.1 mM for prazosin) and stored at − 20 °C until use. U46619 ((Z)-7-[(1*S*,4*R*,5*R*,6*S*)-5-[(E,3*S*)-3-hydroxyoct-1-enyl]-3-oxabicyclo[2.2.1]heptan-6-yl]hept-5-enoic acid) is an agonist of the thromboxane A_2_ receptor (Alexander et al. 2021a) and was dissolved in ethanol. U46619 is commonly used as a thromboxane A_2_ receptor agonist, as thromboxane A_2_ itself is highly unstable (Shen and Tai 1998). Stock solutions (10 mM) were stored at − 80 °C until use. Phenylephrine ((*R*)-3-[-1-hydroxy-2-(methylamino)ethyl]phenol) and methoxamine (α-(1-aminoethyl)-2,5-dimethoxybenzyl alcohol) are α_1_-selective adrenoceptor agonists (Alexander et al. 2021a). Carbachol (carbamoylcholine) and methacholine are muscarinic acetylcholine receptor agonists (Alexander et al. 2021a; Pei et al. 1998). Aqueous stock solutions (10 mM) of noradrenaline, phenylephrine, methoxamine, carbachol, methacholine, and serotonin (5-hydroxytryptamine) were freshly prepared before each experiment. Aqueous stock solutions of endothelin-1 (0.75 mM) and tetrodotoxin (1 mM) were stored at − 20 °C as small aliquots, so that repeating freezing and thawing cycles were avoided. NAV2729, tetrodotoxin, and prazosin were obtained from Tocris (Bristol, UK). U46619 and endothelin-1 were obtained from Enzo Life Sciences (Lörrach, Germany). Noradrenaline, phenylephrine, methoxamine, carbachol, methacholine, and serotonin were obtained from Sigma-Aldrich (Munich, Germany).

### Data and statistical analyses

Data in frequency and concentration response curves are means ± standard deviation (SD), which are presented together with the indicated number (*n*) of independent experiments (see above for design of independent experiments). *E*_max_, pEC_50_, and Ef_50_ values are presented as single values (i.e., means of two technical replicates from double determination, where this was possible) together with means from all independent experiments in scatter plots. Although effect sizes become obvious from frequency and concentration response curves, some effects are additionally reported in the text, as mean differences (MDs) with 95% confidence intervals (CIs), in particular for effects at single agonist concentrations and single frequencies and in order to describe variability in these data sets. Calculation of MD with 95% CI was performed using GraphPad Prism 6. Effect sizes reported in the text were calculated by normalization of values (contractions or *E*_max_ values) obtained with drug to the corresponding control in each single experiment, which were expressed as percentage of corresponding controls and are reported as means with 95% CIs.

Statistical analyses were performed using GraphPad Prism 6. Comparison of whole frequency/concentration response curves was performed by two-way analysis of variance (ANOVA), as explained in detail in an illustrative description (see supplementary information). Our data have paired character, as each vessel was allocated to two groups examined in the same experiment (e.g., DMSO and NAV2729). Consequently, values in corresponding subcolumns (e.g., A:Y1 and B:Y1, see supplementary information) were considered as matched values (but not as repeated measures). Accordingly, the setting “Each column represents a different time point, so matched values are spread across a row” was used, under the menu “Experimental Design” (see supplementary information). Comparisons of contractions at single frequencies or agonist concentrations within curves by multiple comparisons (after two-way ANOVA) were not included, owing to the two-dimensional character and as this is discouraged by the “GraphPad Statistics Guide” (GraphPad Software Inc., San Diego, CA, USA). *E*_max_, pEC_50_, and Ef_50_ values were compared by a paired Student’s *t* test. However, the present study and analyses show an exploratory design and were not designed to test pre-specified statistical null hypotheses (Michel et al. 2020). Besides a lacking hypothesis, typical features of a strictly hypothesis-testing study design were lacking in our study, including a clear preset study plan, blinding, or biometric calculation of group sizes (Michel et al. 2020). Consequently, *p* values reported here need to be considered as descriptive, but not as hypothesis testing (Michel et al. 2020). In line with recent recommendations, the focus was on effect sizes and *p* values were used sparingly (Michel et al. 2020).

Minimum numbers of experiments and group sizes for each series were pre-planned as *n* = 5, to allow the calculation of descriptive *p* values. Data were analyzed, after five or more experiments were performed for a given series. Subsequently, the series was discontinued if it became obvious that no effect could be expected on this basis, or if *p* values were < 0.05 after comparison of frequency/concentration response curves. If these initial results were inconclusive, i.e., pointed to a possible drug effect but without *p* values < 0.05, series were continued and analyzed again. This procedure was possible due to the explorative character, and as long as it is reported in detail (Michel et al. 2020). Flexible group sizes have been in fact recommended for experimental design and analysis in experimental pharmacology, if data are characterized by large variations, what applies here (Curtis et al. 2018; Curtis et al. 2015). Nevertheless, interim analyses were limited to frequency and concentration response curves and did not include *E*_max_, pEC_50_, and Ef_50_ values, which were calculated only after completion of series. No data or experiments were excluded from analyses.

## Results

### High-molar KCl-induced contractions in interlobar and coronary arteries

**Fig. 1:**
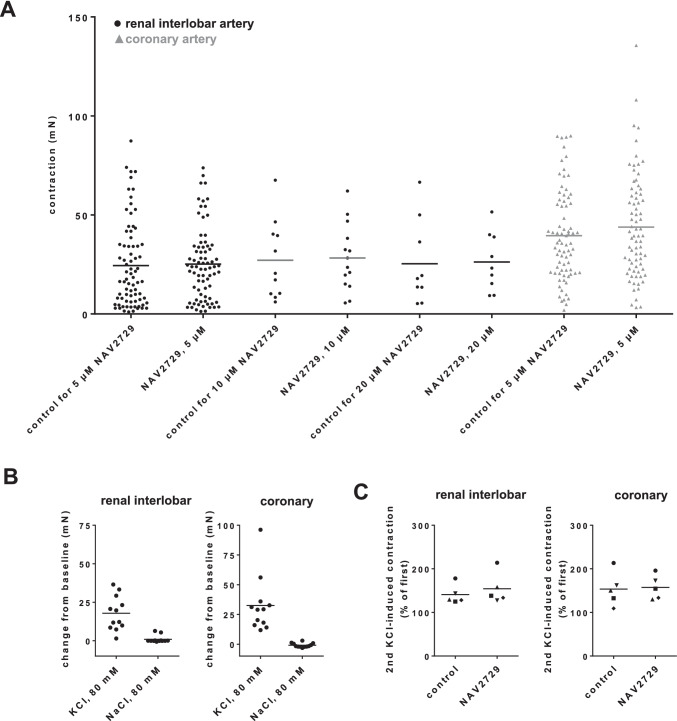
High-molar KCl-induced contractions in renal interlobar and coronary arteries. Contractions by high-molar KCl were induced by the addition of a 2 M KCl solution to normal Krebs–Henseleit solution in organ bath chambers, before construction of frequency and concentration response curves (followed by washout before construction of curves and before the addition of DMSO or NAV2729) (**A**). These KCl-induced contractions were later used for reference of EFS- and agonist-induced contractions, assessed in the same tissues. DMSO was used as solvent for NAV2729 and applied to controls, in amounts required for corresponding NAV2729 in the same experiments. To estimate possible contributions from elevated chloride concentrations due to the hyperosmolar addition of KCl (**B**), vessels were first exposed to additional 80 mM NaCl (by addition of a 2 M solution to normal Krebs–Henseleit solution in organ bath chambers), followed by washout and assessment of KCl-induced contractions as described above. To assess effects of DMSO and NAV2729 on KCl-induced contractions (**C**), contractions by KCl were induced before and after application of DMSO or NAV2729 (5 µM), and the second KCl-induced contraction was expressed as the percentage of the first KCl-induced contraction. Shown are values from each single channel in mN (**A**, **B**), and single values (means from double determinations; paired values obtained from the same tissue and in the same experiment are marked by shared symbols) from each experiment (**C**)

Before construction of frequency response curves by EFS or of concentration response curves for agonists, KCl-induced contractions were elicited by application of a high-molar KCl solution in a hyperosmolar manner to a final potassium concentration of 80 mM, in all vessels and for later reference of EFS- and agonist-induced contractions. Average KCl-induced contractions were similar before application of DMSO (used for controls) and NAV2729 in both vessel types, i.e., in renal interlobar arteries and in coronary arteries (Fig. 1A). To estimate contributions resulting from chloride elevations during hyperosmolar KCl application, effects of 80 mM NaCl (applied in addition to NaCl already existing in Krebs–Henseleit solution) were assessed. Application of 80 mM NaCl did not induce contractions in both vessel types, while KCl-induced contractions were still intact in these tissues (Fig. 1B).

NAV2729 is a small molecule inhibitor with assumed specificity for ARF6 (Yamauchi et al. 2017; Yoo et al. 2016). To assess effects of NAV2729 (5 µM) on KCl-induced contractions, KCl-induced contractions were elicited two times, namely before application of either DMSO or NAV2729 and after application of DMSO or NAV2729 in each tissue of this series. In both vessel types, neither NAV2729 nor DMSO reduced KCl-induced contractions, compared and normalized to KCl-induced contractions assessed before application of DMSO or NAV2729 in the same tissues (Fig. 1C). In both vessel types, KCl-induced contractions were similar after application of DMSO and NAV2729 (Fig. 1C).

### Effects of NAV2729 on EFS-induced contractions of interlobar arteries

**Fig. 2:**
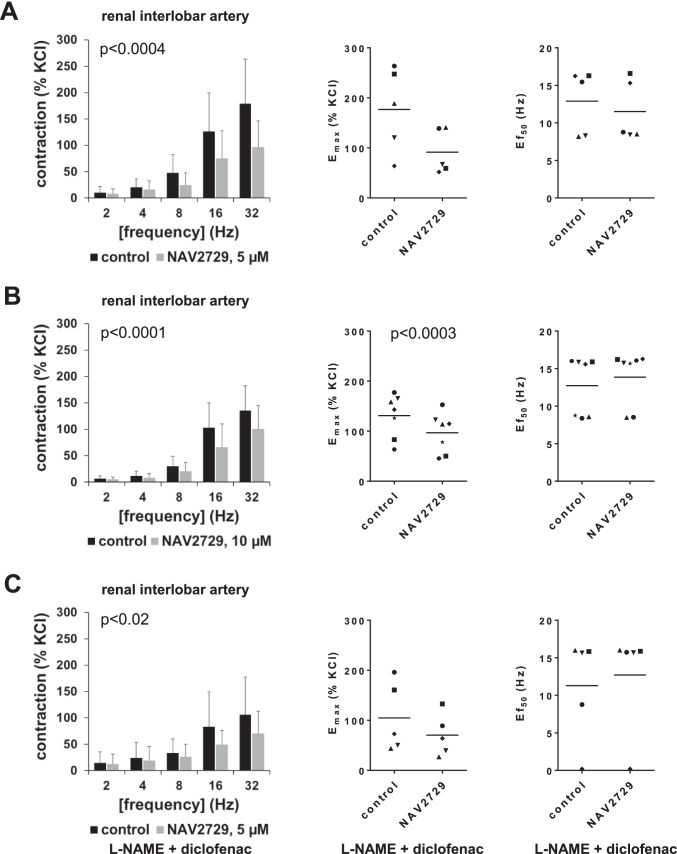
Effects of NAV2729 on EFS-induced contractions in renal interlobar arteries. Contractions were induced by different frequencies of EFS, 30 min following administration of NAV2729 at final concentrations of 5 µM (**A**), 10 µM (**B**), 5 µM in the presence of L-NAME (200 µM, in controls and in NAV2729 groups), and diclofenac (3 µM, in controls and in NAV2729 groups) (**C**), or solvent (controls). Shown are means ± SD in frequency response curves (together with *p* values for whole groups from two-way ANOVA in inserts), and all single *E*_max_ values and frequencies inducing 50% of the maximum EFS-induced contraction (Ef_50_) from all experiments (calculated by curve fitting) in scatter plots (*p* value from paired Student’s *t* test), from experiments using tissues from *n* = 5 animals in **A** and **C** and from *n* = 7 animals in **B**. For each single experiment, tissue from one animal was allocated to the control and the NAV2729 group, which were examined in the same experiment. In scatter plots, paired values obtained from the same tissue and in the same experiment are marked by shared symbols

EFS (2–32 Hz) induced frequency-dependent contractions of interlobar arteries, which were partly inhibited by 5 µM and 10 µM NAV2729 (Fig. 2). If contractions with 5 µM NAV2729 were normalized to corresponding controls in each single experiment, contractions were reduced by 71% (6 to 136) at 8 Hz, by 24% (− 51 to 99) at 16 Hz, and by 41% (13 to 69) at 32 Hz (Fig. 2A). With 10 µM NAV2729, contractions were reduced by 38% (8 to 69) at 8 Hz, by 39% (21 to 57) at 16 Hz, and by 28% (18 to 37) at 32 Hz (Fig. 2B). Accordingly, *E*_max_ for EFS-induced contractions was reduced from 177% (72 to 282) of KCl-induced contraction in controls to 92% (37 to 147) of KCl-induced contraction by 5 µM NAV2729 (MD − 85 percentage points [− 173 to 2]) (Fig. 2A). By 10 µM NAV2729, *E*_max_ was reduced from 131% (91 to 171) of KCl-induced contraction in controls to 97% (60 to 134) of KCl-induced contraction (MD − 34 percentage points [− 44 to − 24]) (Fig. 2B). Ef_50_ values were not changed by NAV2729 (Fig. 2).

To examine a possible involvement of vasorelaxing factors in inhibition of EFS-induced contractions by NAV2729, effects of NAV2729 were examined in the presence of the nitric oxide synthase inhibitor L-NAME and the cyclooxygenase inhibitor diclofenac. An increase in EFS-induced contractions, which may be expected from suppression of nitric oxide and prostaglandin production, did not become apparent from qualitative comparisons of control groups (i.e., EFS-induced contractions without NAV2729) across series with and without L-NAME and diclofenac (Fig. 2A–C).

Partial inhibition of EFS-induced contractions by NAV2729 was still observed in the presence of L-NAME (200 µM) and diclofenac (3 µM) (Fig. 2C). Contractions were reduced by 17% (− 23 to 57) at 8 Hz, by 31% (5 to 58) at 16 Hz, and by 29% (7 to 69) at 32 Hz by 5 µM NAV2729 (Fig. 2C). Accordingly, *E*_max_ values for EFS-induced contractions were reduced from 105% (19 to 191) of KCl-induced contraction in controls to 70% (18 to 123) of KCl-induced contraction (MD − 34 percentage points [− 86 to 17]) (Fig. 2C). Ef_50_ values were not changed by NAV2729 (Fig. 2).

### Effects of tetrodotoxin and prazosin on contractions of interlobar arteries

**Fig. 3:**
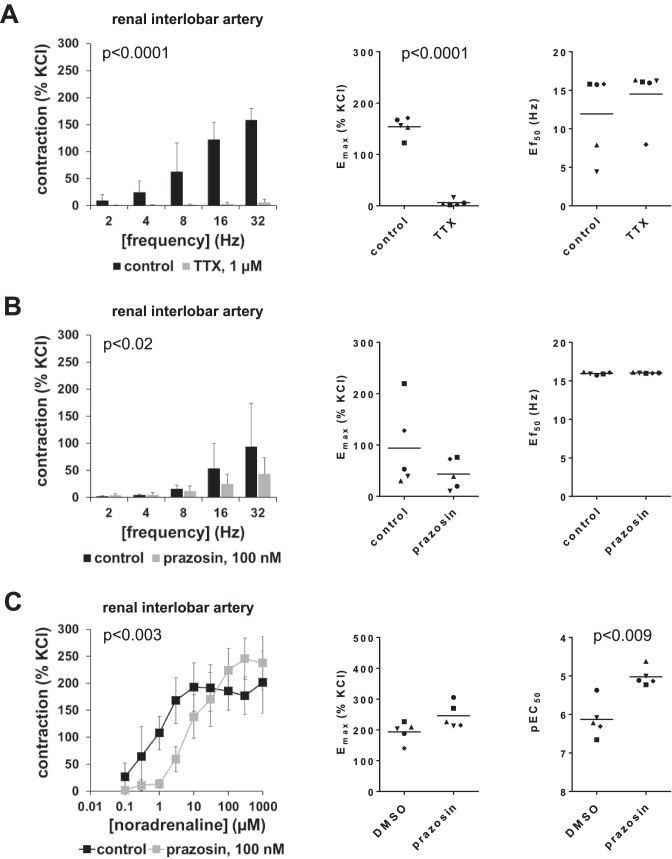
Effects of tetrodotoxin and prazosin on EFS- and noradrenaline-induced contractions in renal interlobar arteries. Contractions were induced 30 min following administration of tetrodotoxin (1 µM) (**A**) or prazosin (100 nM) (**B**) by different frequencies of EFS, or of prazosin (100 nM) by cumulative concentrations of noradrenaline (**C**) in interlobar arteries, or of solvent (controls). Shown are means ± SD in frequency and concentration response curves (together with *p* values for whole groups from two-way ANOVA in inserts), and all single *E*_max_ values and frequencies inducing 50% of the maximum EFS-induced contraction (Ef_50_) or pEC_50_ values for noradrenaline from all experiments (calculated by curve fitting) in scatter plots (*p* value from paired Student’s *t* test), from experiments using tissues from *n* = 5 animals in each series. For each single experiment, tissue from one animal was allocated to the control and the drug group, which were examined in the same experiment. In scatter plots, paired values obtained from the same tissue and in the same experiment are marked by shared symbols

EFS-induced contractions of interlobar arteries were inhibited by tetrodotoxin (1 µM) and partly by the α_1_-adrenoceptor antagonist prazosin (100 nM) (Fig. 3). Tetrodotoxin is a sodium channel inhibitor, which inhibits neurogenic contractions in smooth muscle preparations (Alexander et al. 2021b), while prazosin is a subtype-unselective α_1_-adrenoceptor antagonist (Alexander et al. 2021a). Tetrodotoxin inhibited contractions by 96% (92 to 99) at 8 Hz, by 98% (96 to 99) at 16 Hz, and by 96% (92 to 100) at 32 Hz (Fig. 3A). Accordingly, *E*_max_ values were reduced from 154% (130 to 178) of KCl-induced contraction in controls to 6% (− 1 to 13) of KCl-induced contraction after application of tetrodotoxin (MD − 148 percentage points [− 171 to − 124]) (Fig. 3A). Prazosin reduced EFS-induced contractions by 21% (− 48 to 90) at 8 Hz, by 41% (− 19 to 102) at 16 Hz, and by 43% (− 8 to 95) at 32 Hz (Fig. 3B). Accordingly, *E*_max_ values for EFS-induced contractions calculated by curve fitting were reduced from 94% (− 6 to 194) of KCl-induced contraction in controls to 43% (6 to 81) of KCl-induced contraction after application of prazosin (MD − 51 percentage points [− 121 to 20]) (Fig. 3B). Ef_50_ values were not changed by NAV2729 (Fig. 3A, B).

Noradrenaline induced concentration-dependent contractions of interlobar arteries. Prazosin (100 nM) right-shifted concentration response curves for noradrenaline, resulting in partial inhibition at noradrenaline concentrations from 0.3 to 10 µM, in recovery at higher noradrenaline concentration, and in increased EC_50_ values for noradrenaline, while *E*_max_ values calculated by curve fitting remained unchanged (Fig. 3C). Prazosin reduced noradrenaline-induced contractions by 78% (60 to 96) at 0.3 µM noradrenaline, by 87% (79 to 95) at 1 µM, by 63% (42 to 84) at 3 µM, and by 23% (− 19 to 65) at 10 µM (Fig. 3C). EC_50_ values for noradrenaline were increased by prazosin, reflected by decreases of pEC_50_ values from 6.1 (5.1 to 6.7) in controls to 5.0 (4.7 to 5.1) after application of prazosin (MD − 1.1 [− 0.5 to − 1.7]) (Fig. 3C). *E*_max_ values remained unchanged by prazosin, amounting to 194% (153 to 234) of KCl-induced contractions in controls and to 246% (196 to 296) of KCl-induced contractions after application of prazosin (MD 52 percentage points [− 3 to 108]) (Fig. 3C).

### Effects of NAV2729 on adrenergic contractions of interlobar arteries

**Fig. 4:**
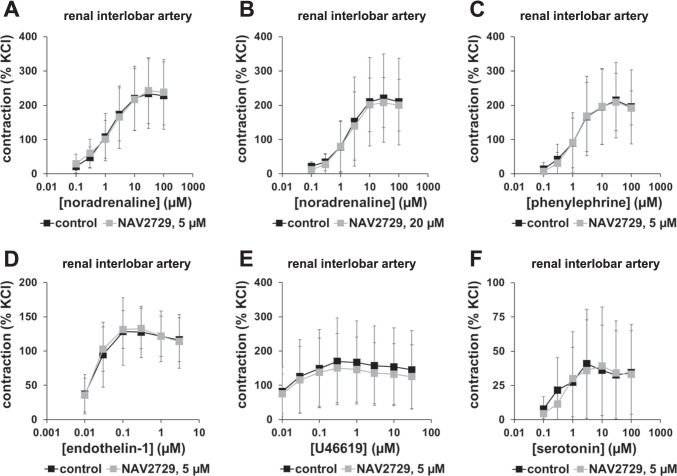
Effects of NAV2729 on agonist-induced contractions in renal interlobar arteries. Contractions were induced by cumulative concentrations of noradrenaline (**A**, **B**), phenylephrine (**C**), endothelin-1 (**D**), U46619 (**E**), or serotonin (**F**), 30 min following administration of NAV2729 (5 µM or 20 µM before noradrenaline, 5 µM before all other agonists) or solvent (controls). Shown are means ± SD in concentration response curves, from experiments using tissues from *n* = 6 animals for noradrenaline and 5 µM NAV2729, *n* = 5 animals for noradrenaline and 20 µM NAV2729, *n* = 6 animals for phenylephrine, and *n* = 5 animals in each series in **D**–**F**. For each single experiment, tissue from one animal was allocated to the control and the NAV2729 group, which were examined in the same experiment

Concentration response curves for noradrenaline remained unchanged after application of NAV2729 using concentrations of 5 µM (Fig. 4A) or 20 µM (Fig. 4B) to interlobar arteries, compared to corresponding controls. Similar to noradrenaline, phenylephrine induced concentration-dependent contractions of interlobar arteries, which remained unchanged by 5 µM NAV2729 (Fig. 4C).

### Effects of NAV272 on non-adrenergic contractions of interlobar arteries

Endothelin-1 (Fig. 4D), the thromboxane A_2_ analog U46619 (Fig. 4E), and serotonin (Fig. 4F) induced concentration-dependent contractions of interlobar arteries, which remained unchanged by 5 µM NAV2729.

### Effects of NAV2729 on agonist-induced contractions of coronary arteries

**Fig. 5:**
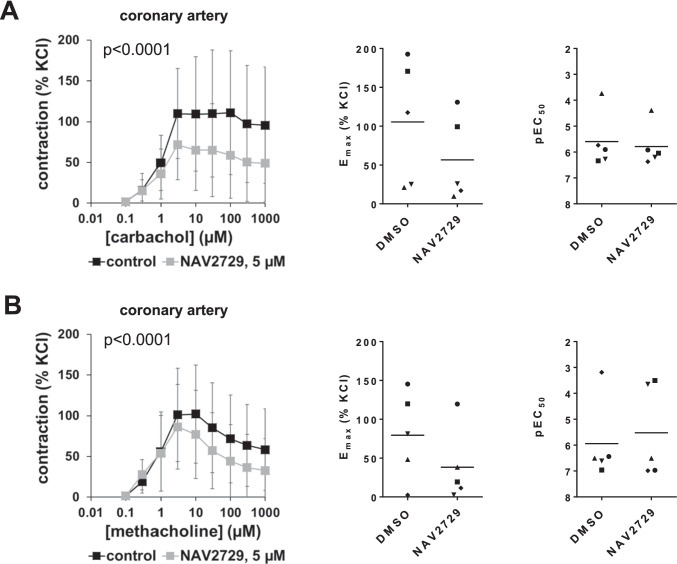
Effects of NAV2729 on cholinergic contractions in coronary arteries. Contractions were induced by cumulative concentrations of carbachol (**A**) or methacholine (**B**), 30 min following administration of NAV2729 (5 µM) or solvent (controls). Shown are means ± SD in concentration response curves (together with *p* values for whole groups from two-way ANOVA in inserts), and all single *E*_max_ values and pEC_50_ values from all experiments (calculated by curve fitting) in scatter plots, from experiments using tissues from *n* = 5 animals for each series. For each single experiment, tissue from one animal was allocated to the control and the NAV2729 group, which were examined in the same experiment. In scatter plots, paired values obtained from the same tissue and in the same experiment are marked by shared symbols

Carbachol and methacholine induced concentration-dependent contractions of coronary arteries, which were partly inhibited by 5 µM NAV2729 (Fig. 5A, B). Carbachol-induced contractions were reduced by 35% (1 to 69) at 3 µM carbachol, by 28% (− 28 to 85) at 10 µM, by 32% (− 16 to 80) at 30 µM, by 43% (9 to 78) at 100 µM, by 41% (− 1 to 83) at 300 µM, and by 41% (− 1 to 83) at 1 mM (Fig. 5A). Accordingly, *E*_max_ values for carbachol-induced contractions were reduced from 105% (6 to 205) of KCl-induced contraction in controls to 57% (− 12 to 125) of KCl-induced contraction after application of NAV2729 (MD − 49 percentage points [− 102 to − 4]) (Fig. 5A). pEC_50_ values for carbachol were not changed by NAV2729 and amounted to 5.6 (4.3 to 6.9) in controls and to 5.8 (4.8 to 6.8) after NAV2729 (MD 0.2 [− 0.3 to 0.7]).

Methacholine-induced contractions were partly inhibited by 14% (− 3 to 30) at 3 µM methacholine, by 25% (0.4 to 49) at 10 µM, by 29% (− 7 to 64) at 30 µM, by 31% (− 25 to 87) at 100 µM, by 33% (− 32 to 98) at 300 µM, and by 35% (− 30 to 100) at 1 mM (Fig. 5B). Accordingly, *E*_max_ values for methacholine-induced contractions were reduced from 79% (9 to 150) of KCl-induced contraction in controls to 38% (− 20 to 97) of KCl-induced contraction after application of NAV2729 (MD − 41 percentage points [− 99 to 17]) (Fig. 5B). pEC_50_ values for methacholine were not changed by NAV2729 and amounted to 5.9 (4.0 to 7.9) in controls and to 5.5 (3.3 to 7.7) after NAV2729 (MD − 0.4 [− 4.1 to 3.2]) (Fig. 5B).

**Fig. 6:**
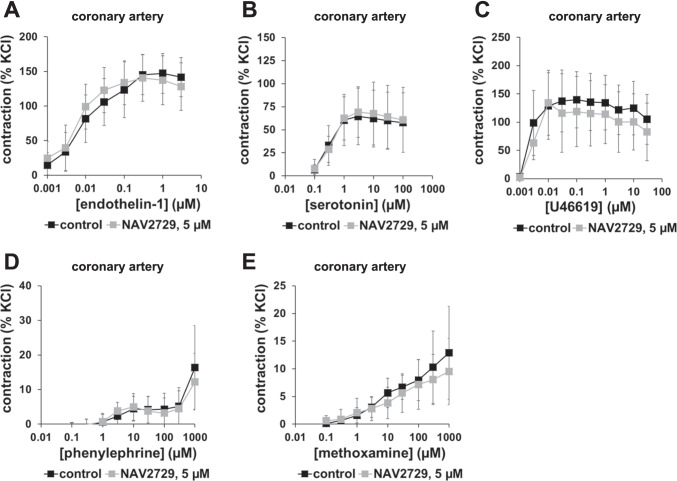
Effects of NAV2729 on agonist-induced contractions in coronary arteries. Contractions were induced by cumulative concentrations of endothelin-1 (**A**), serotonin (**B**), U46619 (**C**), phenylephrine (**D**), or methoxamine (**E**), 30 min following administration of NAV2729 (5 µM) or solvent (controls). Shown are means ± SD in concentration response curves, from experiments using tissues from *n* = 6 animals for U46619 and phenylephrine, *n* = 11 animals for serotonin, and *n* = 5 animals for each other series. For each single experiment, tissue from one animal was allocated to the control and the NAV2729 group, which were examined in the same experiment

Endothelin-1, U46619, and serotonin induced concentration-dependent contractions (Fig. 6A–C). Phenylephrine and methoxamine induced concentration-dependent contractions, without reaching an obvious maximum at the highest applied concentration (1 mM) and without resulting in concentration response curves with sigmoidal character (Fig. 6D, E). None of these contractions was affected by NAV2729 (Fig. 6A–E). Even though two-way analysis of ANOVA pointed to a statistical difference between U46619 curves with and without NAV2729 (see supplementary files), the difference may be too small to conclude a relevant effect of NAV2729 (Fig. 6C).

## Discussion

Vascular smooth muscle contraction takes an essential role in central physiological functions, including regulation of organ perfusion, local blood flow, cardiovascular homeostasis, and blood pressure and is consequently involved in widespread diseases. Accordingly, mechanisms of smooth muscle contraction and drugs for its inhibition are of high potential interest. The findings of the present study suggest that NAV2729, a small molecule inhibitor with previously supposed specificity for ARF6 (Wang et al. 2021; Yamauchi et al. 2017; Yoo et al. 2016), inhibits neurogenic smooth muscle contractions of pig renal interlobar arteries and contractions induced by cholinergic agonists in pig coronary arteries. Contractions by α_1_-adrenergic agonists, serotonin, endothelin-1, or U46619 were not affected by NAV2729 in both vessel types. Assuming that these effects of NAV2729 were imparted by inhibition of ARF6, it may be speculated that ARF6 could be involved in the regulation of renal perfusion and of kidney function by promotion of contractile neurotransmission in the intrarenal vasculature, and in the regulation of cardiac muscle perfusion by promotion of cholinergic vasocontraction of coronary arteries.

NAV2729 has been introduced as an inhibitor of ARF6, which binds directly to ARF6, impeding either its activation by guanosine exchange factors (GEFs) and/or its spontaneous activation independently from nucleotide binding to ARF6 (Yamauchi et al. 2017; Yoo et al. 2016). Consequently, the identity of ARF6-activating GEFS, which may differ between cell types, is not decisive for ARF6 inhibition by NAV2729 (Hongu and Kanaho 2014; Yamauchi et al. 2017). First studies addressing ARF6 inhibition by NAV2729 reported IC_50_ values of 1 µM and 3.4 µM for ARF6 inhibition by NAV2729 in fluorometric and orthogonal radiometric nucleotide exchange assays and suggested no effect on other ARF family members or on other GTPases even at concentrations of 50 µM (Yoo et al. 2016). Effects of NAV2729 on smooth muscle contraction were examined for the first time, after it was observed that secinH3, a presumed inhibitor of cytohesin family GEFs, which belong to the most important ARF6-activating GEFs, inhibited contractions of human prostate smooth muscle (Herlemann et al. 2018). In these studies, NAV2729 inhibited neurogenic and adrenergic contractions of human prostate tissues using a concentration of 5 µM, which was paralleled by inhibition of ARF6, but not of ARF1, RhoA, or Rac1 (Yu et al. 2019). Subsequently, knockout of ARF6 in prostate smooth muscle cells confirmed a procontractile role of ARF6 and suggested a high specificity of NAV2729 at least at concentrations up to 5 µM (Wang et al. 2021). These previous findings (a) prompted us to assume similar effects of NAV2729 on smooth muscle contraction in other organs, including vascular smooth muscle, and (b) suggest that the effects of NAV2729 observed in our current study were mostly, if not exclusively caused by inhibition of ARF6.

Off-target effects of NAV2729 were previously suggested by a study reporting inhibition of ARF1 by 25 µM NAV2729 in nucleotide exchange assays (Benabdi et al. 2017) and by slight effects resulting from 5 to 15 µM NAV2729 in smooth muscle cells with ARF6 knockout (Wang et al. 2021). However, the ARF1 inhibitor brefeldin A did not mimic the effects of 5 µM NAV2729 (Yu et al. 2019). Therefore and considering evidence for lacking off-target effects up to concentrations of 5 µM, off-target effects are probably not involved to relevant extent in inhibition of vasocontraction seen here. Accordingly, applying 10 µM of NAV2729 did not result in larger inhibition of EFS-induced contractions compared to 5 µM, and noradrenaline-induced contractions did not differ after application of 20 µM and 5 µM NAV2729 in interlobar arteries. Generally, vasocontraction may be critically affected by endothelium-derived relaxing factors, including nitric oxide and prostaglandins. The status of the endothelium was not assessed in our study, e.g., by confirming an intact endothelium by acetylcholine-induced relaxations, what may represent a limitation in our study design. In fact, we cannot exclude endothelial damage, due to handling, transport, or preparation, as we did not observe increased EFS-induced contractions after application of L-NAME and diclofenac if contractions in control groups (i. e., with DMSO) are qualitatively compared across the different series. Together, the inhibition of EFS-induced contractions by NAV2729 appeared to be independent from nitric oxide and prostaglandins, as it still occurred in the presence of these inhibitors, at least partly. Finally, we confirmed the neurogenic and adrenergic character of EFS-induced contractions using tetrodotoxin and the α_1_-adrenoceptor antagonist prazosin.

A universal role of ARF6 in promotion of smooth muscle contraction, shared by all contractile receptors or by all or most smooth muscle–rich organs, and as known for RhoA, protein kinase C, or calcium (Somlyo and Somlyo 2003), does not become apparent in the light of our findings. The two vessel types examined here did not show real differences regarding their responses to NAV2729. Rather, they show different characteristics in contractions, as EFS- or noradrenaline-induced contractions do not occur in coronary arteries, while cholinergic contractions typically occur in coronaries but not in interlobar or most other arteries (Li et al. 2021). Accordingly, we examined EFS-induced contractions only in interlobar arteries, and cholinergic contractions only in coronary arteries. NAV2729 inhibited EFS-induced contractions in interlobar arteries, and contractions by muscarinic agonists in coronaries. Inhibition of EFS-induced contractions by NAV2729 occurred by an effect of NAV2729 on a non-α_1_-adrenoceptor part of EFS responses. In both vessel types, NAV2729 did not inhibit contractions by receptor agonists other than those acting on muscarinic receptors. In fact, it has been suggested by studies using other smooth muscle types that contractions in response to muscarinic agonists may differ from those by other receptor agonists. In airway, intestinal, and urinary bladder smooth muscles, β-adrenoceptor agonists exhibit a smaller efficacy and/or potency as relaxing compounds when tissues were contracted by a muscarinic agonist than by any other contractile agent, including KCl (Dale et al. 2014). Thus, mechanisms underlying unique characteristics of muscarinic contractions, and the molecular basis accounting for inhibition of neurogenic contractions by NAV2729 both remain to be identified. Finally, contractions by angiotensin-II, vasopressin, and purinergic agonists may be systemically or locally relevant in the cardiovascular system, but turned out to be weak (i.e., clearly below 100% of KCl-induced contractions) in our recent study using pig interlobar arteries (Li et al. 2021), so that these agonists were not examined here.

Certainly, the physiological relevance of our findings and of a possible role of ARF6 for neurogenic contractions of renal arteries and for cholinergic contractions of coronary arteries remains to be understood. Our study represents a first attempt to address effects of NAV2729 on vascular smooth muscle contraction. The basis to assume such effects were preceding observations that NAV2729 and knockout of ARF6 inhibited contractions of prostate smooth muscle (Wang et al. 2021; Yu et al. 2019). Inhibition of prostate smooth muscle by drugs is an important strategy for medical treatment of lower urinary tract symptoms in benign prostatic hyperplasia, where hypotensive side effects may be limiting and α_1_-adrenoceptors with highest possible selectivity for the α_1A_ subtype are preferred to avoid cardiovascular side effects (Hennenberg et al. 2014; Oelke et al. 2013). Our current findings suggest that effects of NAV2729 in the cardiovascular system could be limited, as we did not observe inhibition of endothelin-1- or thromboxane-induced contractions, which are important vasoconstrictors. Certainly, our findings cannot be automatically generalized to resistance vessels involved in the regulation of blood pressure or of systemic vascular resistance. Even though vessels in our study (having a diameter of 3–5 mm) were small, they were still conductance vessels, but not resistance vessels. Conductance vessels may exhibit a distinct regulation of smooth muscle tone as compared to resistance vessels, while the latter are more relevant for total peripheral resistance. On the basis of our data, however, hypotensive effects of NAV2729 may appear unlikely, as endothelin-1- or thromboxane-mediated contractions are critical for cardiovascular homeostasis but were not inhibited by NAV2729 in both examined vessel types. With all due caution, NAV2729 may emerge to an interesting drug candidate in the context of smooth muscle–based diseases, which shows more or less organ-specific inhibition of smooth muscle contractions. Certainly, addressing effects in other vessel types and in other smooth muscle–rich organs and details about the possible role of ARF6 in smooth muscle contraction merits further investigation.

## Conclusions

NAV2729 inhibits neurogenic vascular smooth muscle contractions in pig interlobar arteries and contractions induced by cholinergic agonists in pig coronary arteries. In both vessel types, NAV2729 does not inhibit contractions induced by receptor agonists other than those acting on muscarinic receptors. Addressing effects in other vessel types and in other smooth muscle–rich organs merits further attention.

## Supplementary Information

Below is the link to the electronic supplementary material.

Supplementary file1 (PZFX 73 KB)

Supplementary file2 (PDF 511 KB)

Supplementary file3 (XLSX 24 KB)

Supplementary file4 (XLSX 261 KB)

Supplementary file5 (XLSX 330 KB)

Supplementary file6 (XLSX 16 KB)

## Data Availability

Original and raw data containing all individual data points are available as supplemental information.
