# Supplementary material for: Inhibition of neurogenic contractions in renal arteries and of cholinergic contractions in coronary arteries by the presumed inhibitor of ADP-ribosylation factor 6, NAV2729
Source: Naunyn Schmiedebergs Arch Pharmacol. 2022 Feb 10;395(4):471–85. doi: 10.1007/s00210-022-02218-2 (PMC8873054; doi:10.1007/s00210-022-02218-2)
Supplement: Supplementary file 2 — Supplementary file2 (PDF 511 KB) [file 210_2022_2218_MOESM2_ESM.pdf]

Supplementary information:

Illustrative description of data analysis and of calculation of p values, by two-way ANOVA using GraphPad Prism 6

(Huang et al., Inhibition of neurogenic contractions in renal arteries and of cholinergic contractions in coronary arteries by the presumed inhibitor of ADP-ribosylation factor 6, NAV2729)

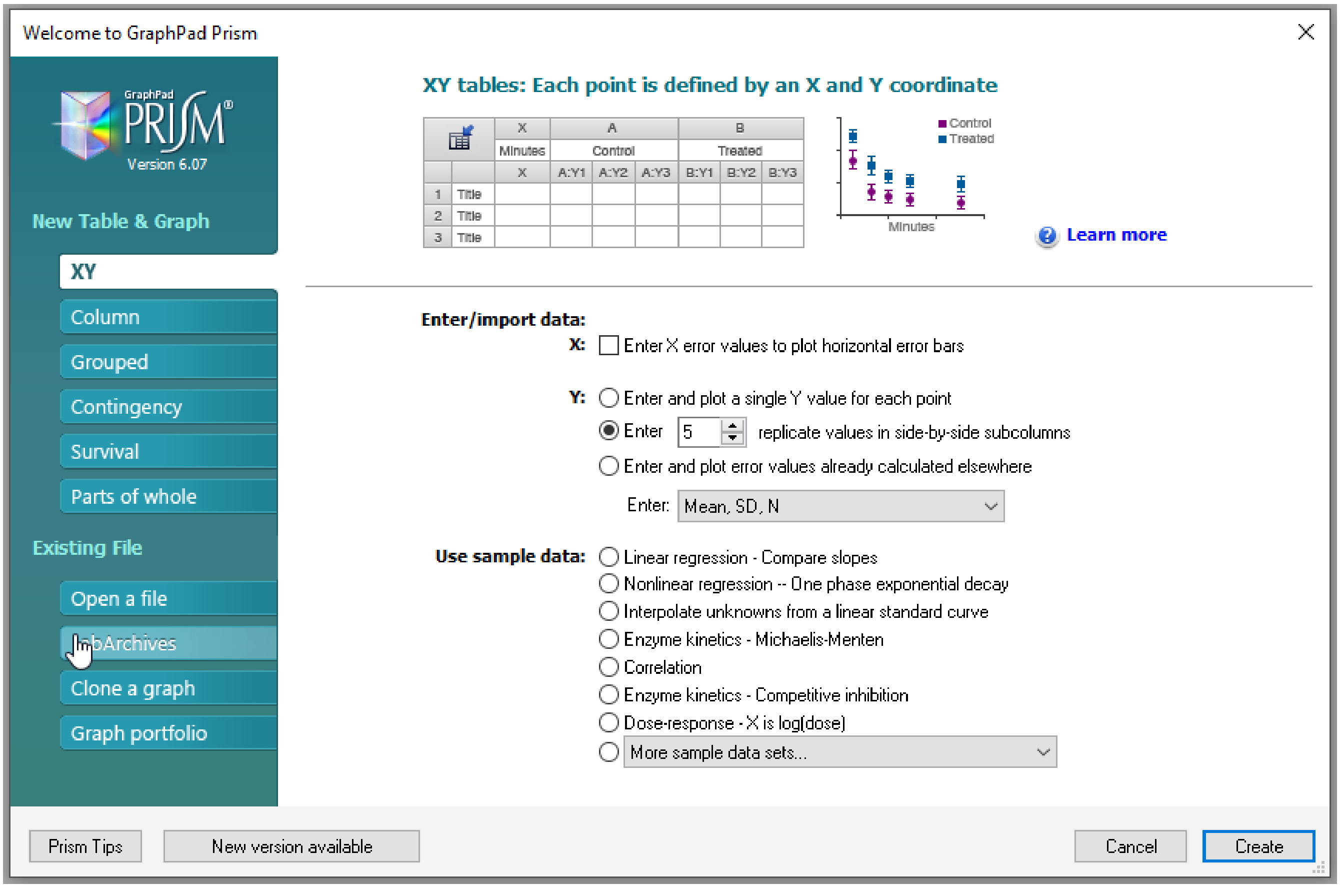

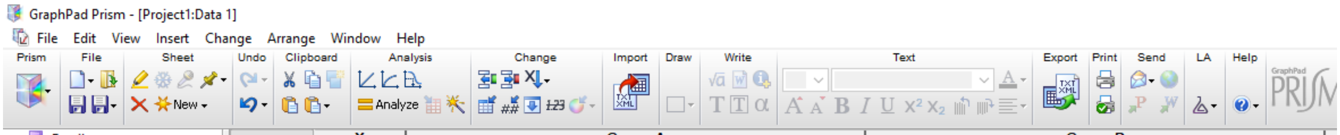

Table format: XY	X	Group A					Group B				
	X Title	Data Set-A					Data Set-B				
	X	A:Y1	A:Y2	A:Y3	A:Y4	A:Y5	B:Y1	B:Y2	B:Y3	B:Y4	B:Y5
1 Title	2	30.126380	10.148110	7.220370	3.923201	1.840818	23.043050	10.212960	9.029228	0.620868	0.247325
2 Title	4	46.305270	19.667760	17.121990	17.607500	0.855352	31.753220	15.005440	35.047240	1.694834	-1.176749
3 Title	8	97.768490	50.210120	51.961600	38.898710	1.619681	54.303230	27.594270	38.990310	6.422259	-0.912591
4 Title	16	180.970800	115.447200	211.124600	97.965900	24.395000	132.859200	63.169430	125.451500	14.549370	43.236460
5 Title	32	196.311600	125.490000	263.493700	247.563100	63.983570	148.699800	71.171100	152.122900	59.303030	51.888660
6 Title											

- Example: data for EFS, -/+ NAV2729, 5 µM
- Horizontal = "row"
- Vertical = "column"

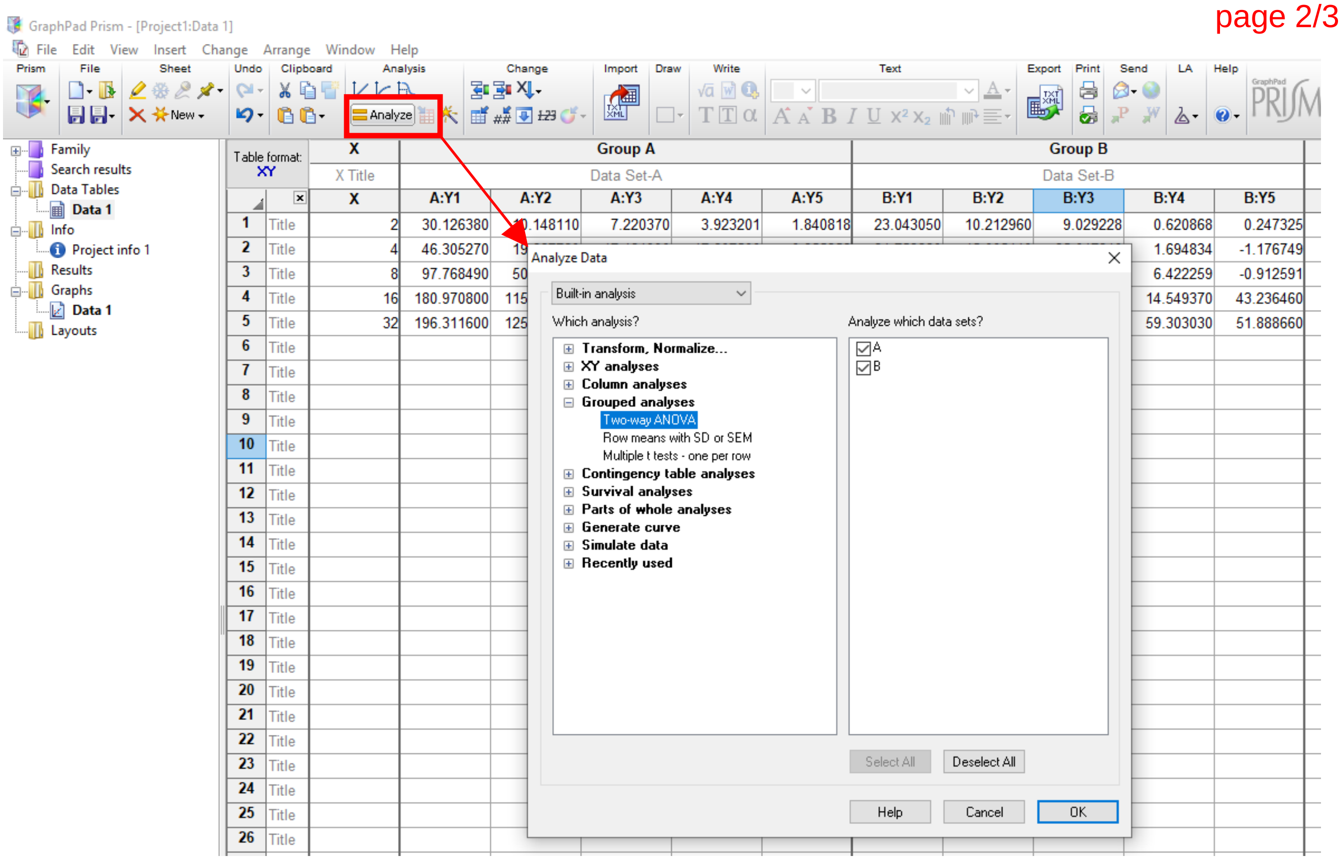

Analyze → Grouped analyses → Two-way ANOVA → OK

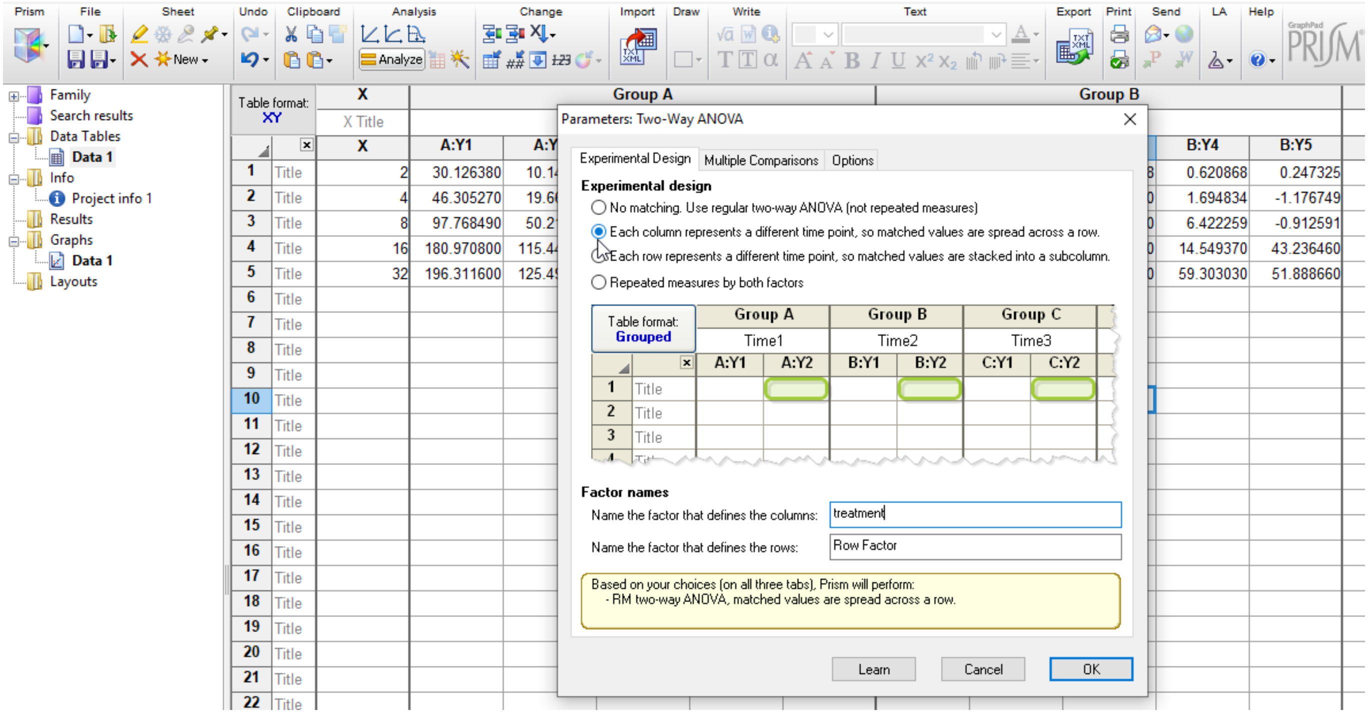

Menue "Experimental design":

- Choose 2nd option (as indicated, according to the paired character of the experimental design)
- Name of column factor: "treatment" (= control or NAV2729, prazosin, etc.)

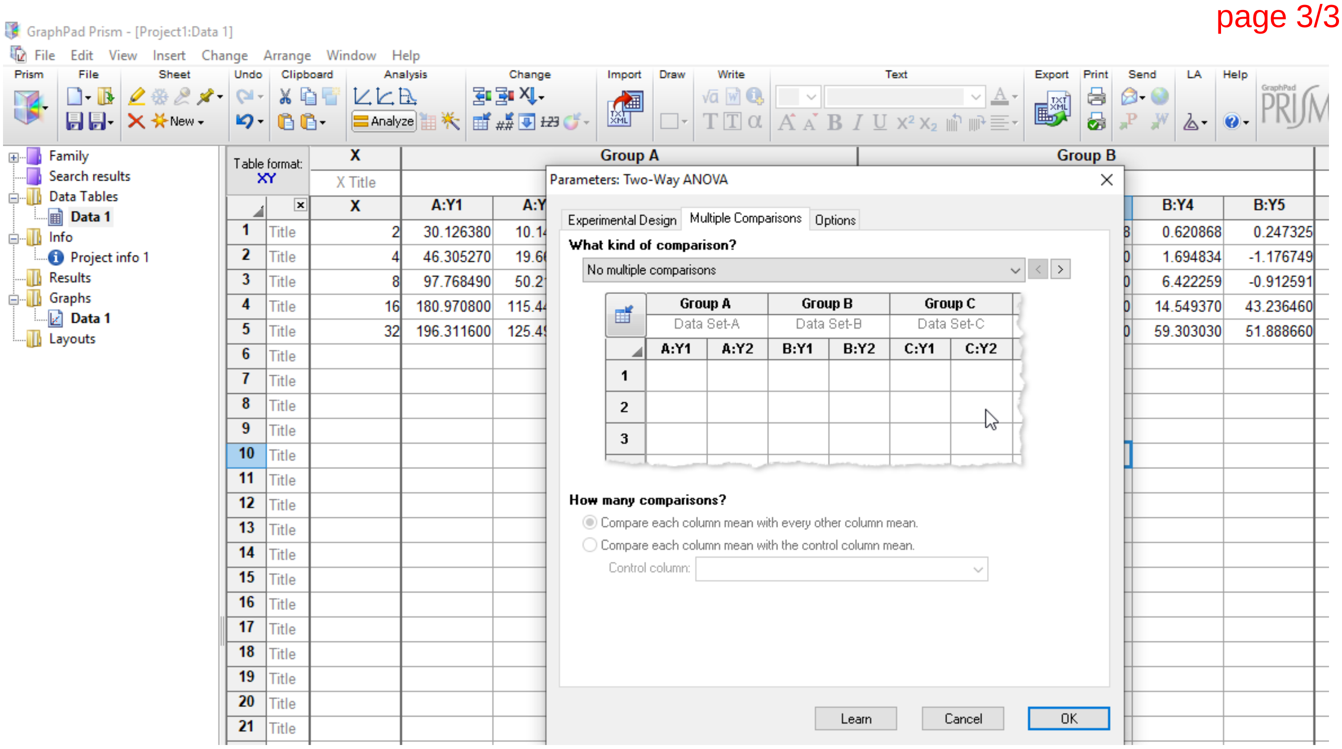

Menue „Multiple Comparisons“:

- Choose "No multiple comparisons"
- Menue "Options": unchanged, nothing
- OK

↓

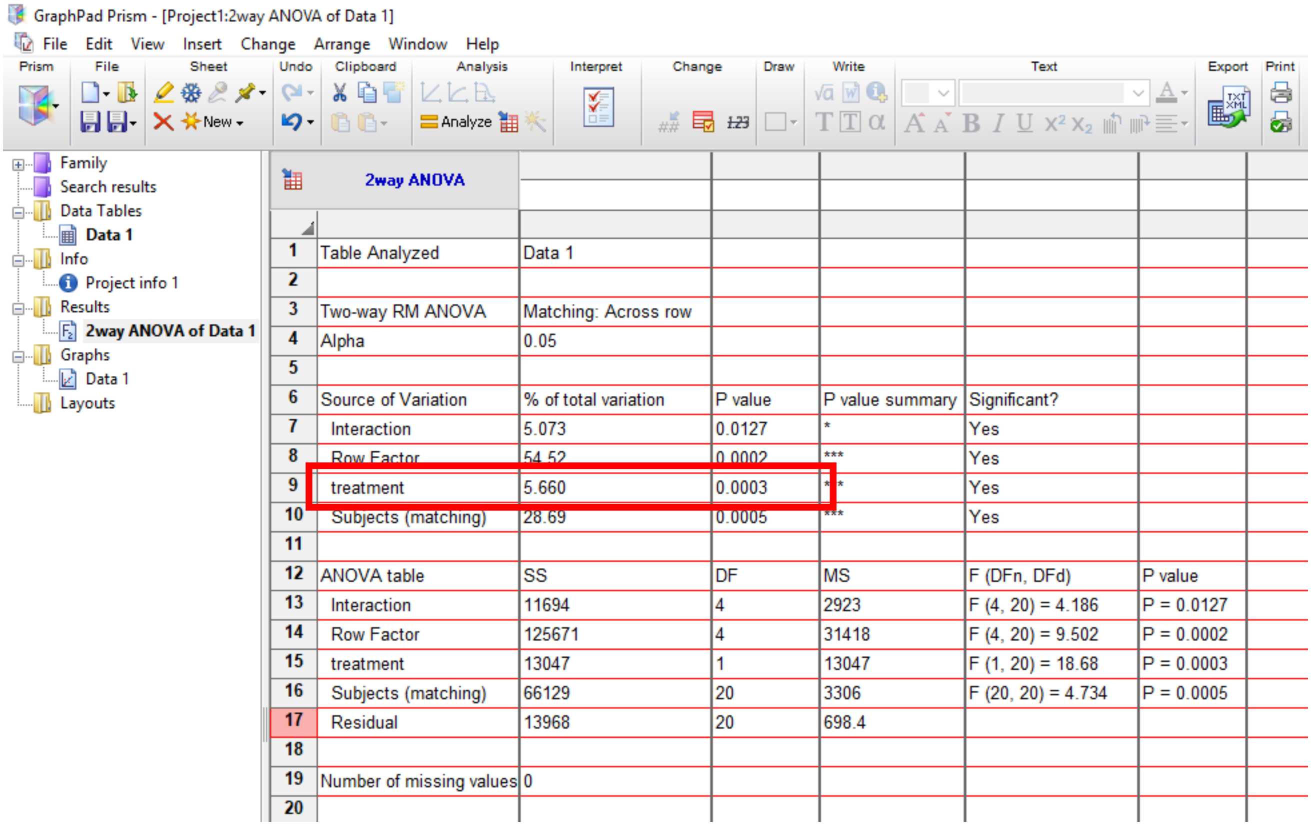

1	Table Analyzed	Data 1				
2						
3	Two-way RM ANOVA	Matching: Across row				
4	Alpha	0.05				
5						
6	Source of Variation	% of total variation	P value	P value summary	Significant?	
7	Interaction	5.073	0.0127	*	Yes	
8	Row Factor	54.52	0.0002	***	Yes	
9	treatment	5.660	0.0003	***	Yes	
10	Subjects (matching)	28.69	0.0005	***	Yes	
11						
12	ANOVA table	SS	DF	MS	F (DFn, DFd)	P value
13	Interaction	11694	4	2923	F (4, 20) = 4.186	P = 0.0127
14	Row Factor	125671	4	31418	F (4, 20) = 9.502	P = 0.0002
15	treatment	13047	1	13047	F (1, 20) = 18.68	P = 0.0003
16	Subjects (matching)	66129	20	3306	F (20, 20) = 4.734	P = 0.0005
17	Residual	13968	20	698.4		
18						
19	Number of missing values	0				
20						

p<0.0004 (as indicated in figure 2A)
